# Photo-generated metamaterials induce modulation of CW terahertz quantum cascade lasers

**DOI:** 10.1038/srep16207

**Published:** 2015-11-09

**Authors:** Francesco P. Mezzapesa, Lorenzo L. Columbo, Carlo Rizza, Massimo Brambilla, Alessardro Ciattoni, Maurizio Dabbicco, Miriam S. Vitiello, Gaetano Scamarcio

**Affiliations:** 1 Dipartimento Interateneo di Fisica, Università degli Studi e Politecnico di Bari, via Amendola 173, I-70126 Bari Italy; 2 CNR-IFN UOS Bari, via Amendola 173, I-70126 Bari Italy; 3 Dipartimento di Scienza e Alta Tecnologia, Università dell’Insubria, via Valleggio 11, I-22100 Como Italy; 4 CNR-SPIN, via Vetoio 10, I-67100 L’Aquila Italy; 5 NEST, CNR - Istituto Nanoscienze and Scuola Normale Superiore, piazza San Silvestro 12, I-56127 Pisa Italy

## Abstract

Periodic patterns of photo-excited carriers on a semiconductor surface profoundly modifies its effective permittivity, creating a stationary all-optical quasi-metallic metamaterial. Intriguingly, one can tailor its artificial birefringence to modulate with unprecedented degrees of freedom both the amplitude and phase of a quantum cascade laser (QCL) subject to optical feedback from such an anisotropic reflector. Here, we conceive and devise a reconfigurable photo-designed Terahertz (THz) modulator and exploit it in a proof-of-concept experiment to control the emission properties of THz QCLs. Photo-exciting sub-wavelength metastructures on silicon, we induce polarization-dependent changes in the intra-cavity THz field, that can be probed by monitoring the voltage across the QCL terminals. This inherently flexible approach promises groundbreaking impact on THz photonics applications, including THz phase modulators, fast switches, and active hyperbolic media.

Quantum cascade lasers (QCLs) are the best performing semiconductor sources operating in the mid-infrared range, at wavelengths λ > 6 μm, and at terahertz (THz) frequencies^1^ and can be actively modulated up to tens of gigahertz^2^. A complementary approach for the external modulation of THz beams relies on the use of metamaterials^3^ or meta-atoms^4^, whose sub-wavelength patterns are engineered to produce a resonance in the optical response, while the electric field amplitude is efficiently confined in specific regions of space. Beside static metamaterials^5^,^6^, reconfigurable all-optical structures^7^,^8^,^9^,^10^,^11^,^12^,^13^, whose effective medium response can be actively tuned, disclose the potential to devise active THz modulators. This emerging technology intrinsically provides versatile and reconfigurable optical response, when a spatially structured optical beam creates electron-hole pairs in the semiconductor it impinged on, thereby ensuing photo-created metamaterials induced by alternating metallic and dielectric regions without need for microfabrication. Furthermore, it offers the advantage of gray-scale lithography^14^,^15^, allowing for a gradual variation of the complex permittivity as a function of the pump optical power, and enormous flexibility in the artificial design of THz effective medium response. Recently, an optical transient metamaterial has been employed to demonstrate ultrafast modulation of the polarization state of the broadband THz wave packets emitted by a femtosecond mode-locked laser^16^.

Here, we demonstrate manipulation of THz QCLs emission properties by tailoring the re-injected optical feedback from steady birefringent metamaterials photo-designed onto homogeneous semiconductor surfaces. Specifically, we use back-reflections from a set of reconfigurable photonic structures consisting of optically generated metal-like gratings, having a period much shorter than the THz wavelength, to significantly affect the QCL state, such as the compliance voltage, emitted power and wavelength, all exhibiting very high sensitivity to optical re-injection^17^. We exploit the inherent coherence of optical feedback interferometry to characterize the complex refractive index of the all-optical THz metamaterials in a contact-free and detector-less configuration. The experimental data are validated via a theoretical approach based on i) the Drude model and the effective medium theory^5^, which predicts the complex permittivity induced by the sub-wavelength alternance of dielectric and metallic parts onto a semiconductor; ii) the Lang-Kobayashi model^18^, which accounts for the QCL response under optical feedback.

The significant anisotropy of the semiconductor reflectance induced by stationary photonic patterns opens new possibilities to simultaneously attain negative or positive permittivity for the two field polarizations^14^. This offers a concrete perspective for devising photo-induced THz hyperbolic metamaterials, an important prerequisite for achieving hyperlensing^5^, or nonlinear metamaterials, leading to subwavelength soliton formation^19^. By eliminating the need for complex and time-consuming fabrication processes, the realization of a novel class of all-optical, smart and high-performance metadevices raises the prospect for effective control of THz QCL emission properties and functionalities.

## Results

### Experiment

**Figure 1:**
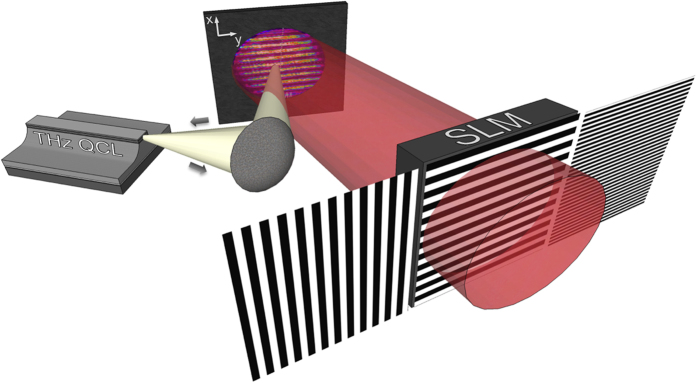
Schematic for optically-driven manipulation of THz QCL. A spatial light modulator (SLM) finely alters the intensity profile of a NIR laser beam to irradiate the surface of a semiconductor slab and tailor its local THz response. The photo-induced density of free-electron plasma onto the 1-mm-thick slab of *n*-type silicon is spatially reconfigurable, thereby producing permittivity modifications in a thin film at the surface (i.e. modulation depth ≈ 13 μm with an 832 nm excitation) which effectively modify the emission properties of THz QCL in the self-mixing configuration. Sub-wavelength intensity patterns are translated into peculiar distributions of the semiconductor refractive index, thus inducing THz QCL field modulation.

To design an albeit complex reflectivity of semiconductors in the THz frequency range, we create all-photo-induced metamaterials by controlling the intensity profile of an optical pump beam in the sub-wavelength scale, according to the Drude theory^5^. Specifically, Fig. 1 shows the cross-section of a near-infrared (NIR) continuous-wave (CW) laser beam (i.e. λ_*NIR*_ = 0.832 μm) being structured through a spatial light modulator (SLM), in order to generate several patterned photo-carriers distributions onto a semi-insulating semiconductor wafer (see Methods for details). Thus, the optical reflectivity of the photo-designed semiconductor surface is probed by the THz radiation emitted by a QCL. The latter, a resonant-phonon single-mode QCL^20^, works in the self-mixing (SM) configuration^18^, with its THz beam, a plane wave with wavelength λ_*THZ*_ = 76.3 μm (3.93 THz), being normally incident to the semiconductor surface and polarized in the x-y plane in Fig. 1.

Coherent feedback interference, or self-mixing phenomena in QCLs, are well exploited in a number of applications^21^,^22^,^23^,^24^, and here depend on the relative phase difference between the field of the solitary laser and the field back-reflected from the THz metamaterials artificially created on the semiconductor surface. Accordingly, the modifications of the voltage (Δ*V*) at the QCL terminals measure the effective response of the dielectric properties to the modulation induced by the spatial distribution of the photo-excited carriers onto the semiconductor. These voltage modifications, as detected by a lock-in amplifier (see Methods), are proportional to the variation of the QCL carrier density (Δ*N* ) with respect to the value at the solitary laser threshold^18^,^25^:


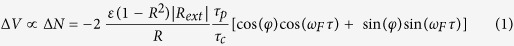


where 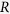 is the laser facet reflectivity taken entirely real without loss of generality, and 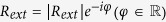 is the semiconductor slab reflectivity affected by the complex permittivity of the optically induced metamarial; 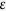 accounts for the coupling losses in the external cavity between the QCL and the slab; 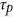 and 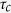 are the photon and the non-radiative carrier lifetimes and 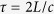 where *L* is the external cavity length.

The CW laser frequency 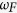 is accounted for the description of the optical feedback effect on the laser dynamics as a solution of the following transcendental equation:


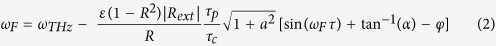


where 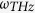 is the solitary laser angular frequency and *α* is the linewidth enhancement factor.

### Reconfigurable all-optical THz modulator

The metamaterial structure consists of a series of computer-generated arrays with equally spaced NIR illuminated stripes of period 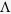 and duty cycle of 50%, as sketched in Fig. 1. Band-to-band absorption in the semiconductor creates a grating of free electrons and holes, yielding polarization-dependent reflectivity and phase-shift at the semiconductor surface, both of which change the feedback interferometric conditions when the THz field is reflected back into the QCL.

**Figure 2:**
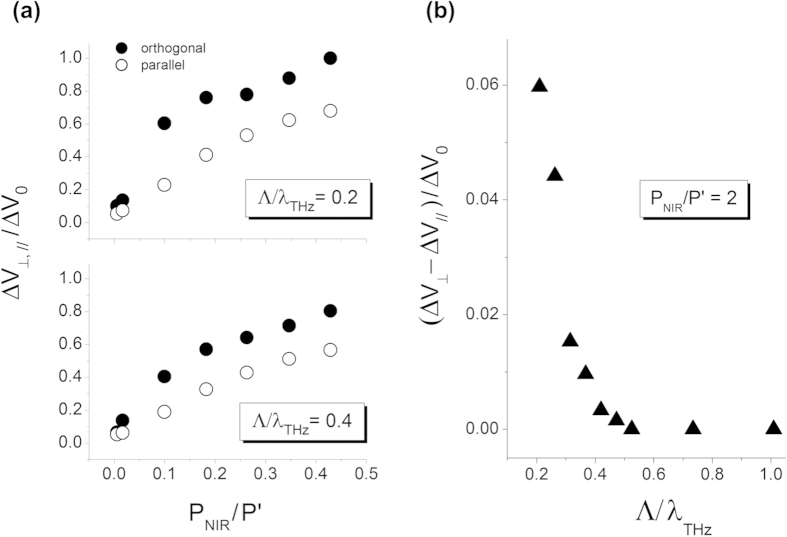
(**a**) Gray-scale lithography. THz-QCL voltage modulation as a function of the power density of the NIR laser beam. The normalization values are: P′ = 35 mW/cm^2^, the power density at transparency; Δ*V*_0_ = 80 *mV*, the maximum voltage corresponding to fully developed constructive interference between the QCL cavity field and back-reflected field. Here, λ_THz_ ≈ 76.3 μm and the excited volume fraction is constant with respect to period Λ of the photo-designed grating on the silicon slab, as measured by replacing the latter with a CMOS camera (pixel size 4.4 × 4.4 μm). A set of 90°-rotated beam profiles, having the stripe direction perpendicular (solid symbols) and parallel (open symbols) to the QCL polarization, is compared for two representative grating periods, respectively. (**b**) THz modulation effect. The voltage modulation 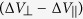 as a function of Λ monotonically increases in the explored sub-wavelength region, being Λ_min_ ~ 15 μm (i.e. Λ ≈ λ_THz_/5) the minimum period achievable with our setup.

Figure 2a shows the dependence of the laser voltage (i.e. of the carrier density) on the normalized power density of the sub-wavelength pattern photo-designed onto the silicon surface (i.e. the electron-hole plasma density). We collect the signal by rotating the patterns of the NIR laser beam by 90°, for two representative grating periods having, alternatively, the stripe direction orthogonal (Δ*V*_⊥_) and parallel (Δ*V*_||_) to the QCL polarization. The degree of artificial anisotropy can be induced either by changing the period of the stripes or by continuously tuning the NIR photo-induced excitation (i.e. gray-scale lithography), thus offering enormous flexibility for tuning of the macroscopic permittivity. Two THz plane waves experience a different reflectance if linearly polarized along or perpendicular to the stripes, and the resultant amplitude of the optical feedback field interfering within the QCL active medium changes accordingly, as well the voltage at the QCL terminals does. Particularly, the maximum voltage modulation of about 25 mV ((Δ*V*_⊥_ − Δ*V*_‖_)/Δ*V*_0_ ~ 0.3 in Fig. 2a) corresponds to a maximum QCL power modulation of about 0.4 μW. This value, obtained considering the optical power and electrical characteristics of the investigated THz QCL, should be compared to the range of emitted optical powers (up to a few microwatts) at the operating currents slightly above threshold where the sensitivity to the optical feedback is maximum (see Methods).

The different complex refractive index experienced by the 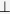 or 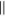 field can be described as an effective induced homogeneous birefringence switched on by the NIR photo-excited carriers onto the semiconductor surface, and increases by reducing the grating period in the sub-wavelength regime, despite the effectively excited volume fraction remaining constant.

In order to isolate purely geometrical effects from accidental inhomogeneities of the NIR pump, we plot in Fig. 2b the residual birefringence measured at P_NIR_ well above the saturated power density, where stationary carrier distribution can be safely assumed. Specifically, Fig. 2b shows the pure effect of the period of such artificial gratings on the THz response of the silicon slab. The artificial birefringence, here measured as induced voltage change 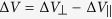, is effective in the sub-wavelength regime, i.e. at a periodicity smaller than the vacuum wavelength of the THz radiation, where the metamaterials forms a homogeneous anisotropic crystal^14^, as predicted by the effective medium theory. Finally, vanishing of the photo-induced birefringence at 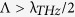 allows us to exclude that binary diffraction grating effects as those described in ref. 26 are at the origin of the observed anisotropy.

### Theoretical analysis

To highlight the relation between the response of photo-excited metamaterials in the THz frequency range, and the QCL performance detected by the self-mixing interferometry, we provide a theoretical validation of the experimental evidence by considering the configuration illustrated in Fig. 1. We apply the Drude model and the effective medium theory to calculate the variation of the complex reflection coefficient R_*ext*_ of a silicon wafer with respect to the intensity of the pump NIR beam and the incident QCL polarization. Thus, we estimate the associated variation of the self-mixing signal using Eqs (1) and (2).

In presence of a NIR beam illuminating a Si wafer under normal incidence, with a spatial intensity distribution given by


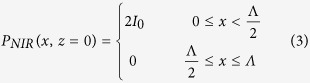


the semiconductor optical response at the THz frequency, 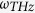, can be locally described by the complex permittivity^5^,^27^:


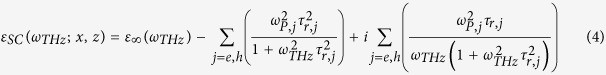


where 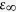 is the background dielectric permittivity; 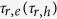 is the free electrons (holes) relaxation time (i.e. average collision time). The plasma frequency 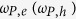 depends on the free electrons n (holes p) density through the relation 
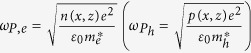
, where 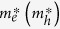 is the electron (hole) effective mass, *e* is the electron charge and 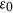 is the vacuum permittivity.

In the limit of pump intensity P_*NIR*_ well below transparency, we assume a linear dependence of the carrier density n and p from the near-infrared field propagating inside the semiconductor slab, consistently with the results reported by Kamaraju *et al.*^16^:


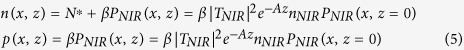


where N^*^ is the carrier density in the beam-blanked sample; *A* is the unsaturated absorption; 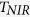 and 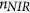 are the near-IR transmission coefficient and refractive index, respectively; *β* is a fitting parameter related to the carrier-to-power density ratio at transparency.

As predicted by the effective medium theory (see Eqs. (4.4) and (4.7) in ref. 5), the semiconductor slab behaves like a homogeneous anisotropic crystal when 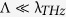, with the effective permittivity for parallel and orthogonal polarized THz plane wave given respectively by (see Supplementary Discussion 1):


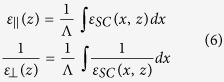


We used Eq. (6) to calculate the complex reflection coefficients 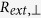 and 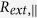. and study the influence of spatially modulated optical intensities on the semiconductor response at the THz frequency (see Supplementary Discussion 2).

We get:


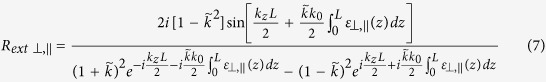


where 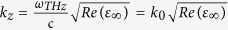 and 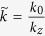


**Figure 3:**
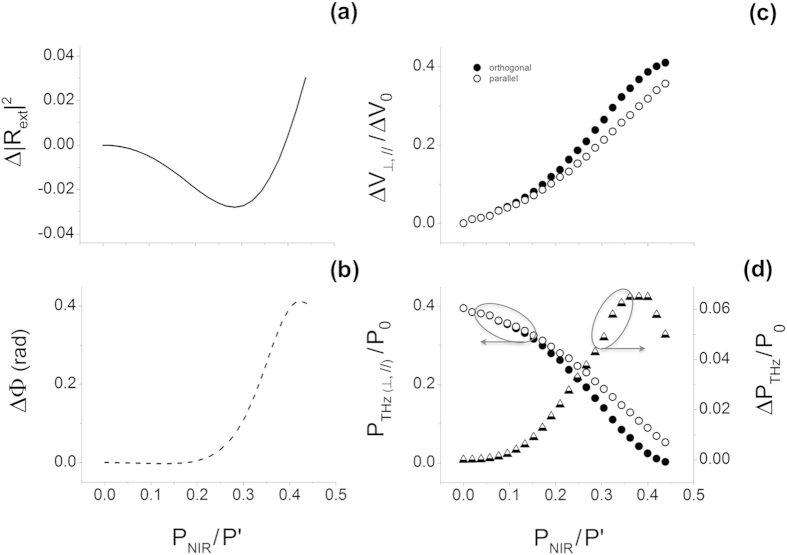
Analysis of Lang-Kobayashi model with target anisotropy predicted by the effective medium theory. Panel (**a**,**b**): Variation with the normalized power density of the optically induced difference in the modulus (Δ|R_ext_|^2^) and phase (ΔΦ) of the Si reflection coefficient for orthogonal and parallel polarization (see definition in the text). Panel (**c**,**d**): Corresponding THz QCL voltage change and emitted power density. The latter is normalized on the maximum value P_0_ at the fully developed constructive interference. The difference between emitted power in case of perpendicular and parallel polarization (ΔP_*THZ*_) is also shown [triangles in panel (**d**)]. The grating period is assumed Λ ≪ λ_*THZ*_. Parameters have been taken from literature^24^,^27^. The values of the other parameters used in simulations are: τ_r,e_ = τ_r,h_ = 3.23 × 10^−13^ s; m_e_* = 0.27m_0_ and m_h_* = 0.37m_0_ (m_0_ being the electron mass); N^*^ = 10^18^ m^−3^; ε_∞_ = 11.7+i0.01; Α = 800 cm^−1^; T_NIR _= 0.45; n_NIR_ = 3.4; β = 1.25 × 10^21^ m^−1^W^−1^, τ_c_ = 37.4 × 10^−12^ s; τ_p_ = 32.4 × 10^−12^ s; W = 1.5; α = 1.5; Σ = ε(1 − R^2^)/R = 0.03.

Figure 3a,b show the effect of the optically induced anisotropy versus the normalized pump power (P_NIR_/P ′) in terms of variation of the modulus 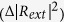 and of the phase (ΔΦ) of the reflectivity coefficient of the silicon wafer, here calculated using Eq. (7) and given, respectively, by:


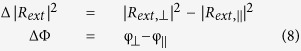


A maximum value of 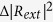 and 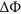 around 3% and 0.4 rad, respectively, is calculated for a relatively low value of the pump power, i.e. at 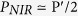. Also, note that interference effects due to the finite thickness of the Si slab are explicitly accounted for: we verified that quasi-periodical results occur in Eq. (7) with a periodicity of 
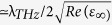
.

Figure 3c shows the corresponding voltage change at the QCL terminals calculated by inserting the coefficients given by Eq. (7) in Eqs (1) and (2). A monotonic increase of the QCL voltage with respect to the NIR excitation is predicted for the two polarizations up to 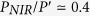, where the relative difference reaches about 15% of the maximum Δ*V* value obtained for stripes orthogonal to the THz polarization, in good agreement with the experimental results given in Fig. 2a.

Finally, to estimate the effect of the photo-induced subwavelength gratings on the emission properties of the THz QCL, we calculate the normalized difference between the estimated QCL power (*P*_*THZ*_) for parallel and perpendicular polarization, as plotted in Fig. 3d. In the framework of the Lang-Kobayashi model^18^:


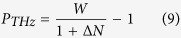


where 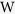 is the electrical pump power normalized to its value at the free running laser threshold. These results demonstrate that, analogously to what was proposed by Rakic *et al.*^28^, self-mixing interferometry provides a powerful tool to detect both amplitude and phase modulations, here all-optically induced onto the semiconductor surface, proving its intrinsically high sensitivity to recording fine modifications of the complex refractive index. Moreover, the good agreement between theoretical models and experimental data provide an effective way to control and manipulate the QCL emission properties through reconfigurable THz metamaterials. This opens to innovative realizations of electromagnetic response of semiconductors in the THz domain, among which the hyperbolic behaviour recently predicted by Rizza *et al.*^14^. The discrepancy between theory and experiment may be explained by considering the dielectric homogenization (that fully justifies the use of the effective medium approach) being only partially achieved in the experiment, where the minimum value of 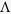 is 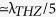. Contributions due to spatial nonlocality may also play a role^29^. Other effects, i.e. the THz wave diffraction inside the semiconductor (i.e. finite size of the THz beam) and the free-carriers diffusion, the latter being strongly sample-dependent^27^, are not taken into account for sake of simplicity in our semi-analytical calculation, and would only imply a correction of the free-carriers variation with the pump intensity.

## Discussion

We provide experimental demonstration of the possibility to devise a stationary, optically-induced metamaterial in the THz frequency domain and support our results with a specifically developed theoretical model. The capability to modulate the emission properties of a continuous wave QCL through optical feedback effects provided by metamaterial reflectors is shown. This is achieved in real-time, as opposed to transient effects in pulsed regimes, providing a mean to customize the field amplitude and phase emitted by the THz QCL. The effect relies on purely optical-carrier coupling, not resorting to intrinsically slow and nonlocal effects such as thermal modulations^30^. Therefore, very differently from power modulations induced via external current modulations, this effect is achieved without significantly altering the spectral properties of the emitter, thus adding a new degree of freedom to the control of THz QCL emitters.

The demonstrated concept paves the way to novel optical applications in the THz frequency range: photonic metastructures can be optically tailored at the micron scale, allowing to access more sophisticated geometries. Even with the simple stripe patterning, semiconductor may exhibit hyperbolic metamaterial properties^14^ that could be exploited to device flat reconfigurable optics (polarizers, switches, modulators, …) and metamaterials (optical antennas, hyperbolic media, …).

## Methods

### Experimental setup

The free electron plasma is induced by a CW *pump* beam at 832 nm delivered by a GaAlAs laser diode (Hitachi HL8325G) on a bare Silicon wafer. The latter is a *n*-doped high resistivity (30 kΩ∙cm ) float zone silicon (HRFZ-Si) produced by Tydex Company, with a diameter of 50.8 mm and nominal thickness of 1 mm (transmittance ≈55% to the THz beam^24^). Assuming that the photo-generated carriers are confined in the absorption depth at 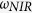 (i.e., a thin layer of length ≈ 13 μm), the NIR beam, passing through a spatial light modulator (SLM), generates arbitrarily patterned carrier density distributions on the semiconductor surface which are *probed* using a QCL in the self-mixing configuration, i.e. subject to optical reinjection from the silicon. This scheme combines the local oscillator, mixer and detector all in a single chip. The QCL, emitting at a frequency around 3.93 THz, is housed in a liquid helium continuous-flow cryostat fitted with a polymethylpentene window, and kept at a fixed heat sink temperature of 15 K. To maximize the sensitivity to coherent optical feedback, the QCL is driven near threshold (i.e., I_th_ ≈ 650 mA for the solitary QCL) at a constant current I = 700 mA for CW mode operation. The THz beam is collimated using a 90° off-axis parabolic mirror with an equivalent focal length of 25.4 mm and focused by a second reflector of F-number 2 at normal incidence onto the semiconductor slab. A wire-grid polarizer ensures THz polarization control. The reflected radiation is collected by the same parabolic mirrors and coupled back into the laser cavity, producing voltage modulations on the laser terminals at the current controller (LDX-3232), which are fed to the AC-coupled signal input of a lock-in amplifier referenced by a mechanical chopper.

### Real-time analysis of the interferometric signal

Photo-induced modifications of the semiconductor permittivity and real-time tuning of its birefringence with CW patterned intensity profiles in the sub-wavelength scale effectively affect the ultrafast response of THz-QCLs in the self-mixing configuration, ultimately limited by the carriers lifetime (~ps), and the corresponding compliance voltage at the laser terminals. The homodyne (coherent) nature of the proposed experiment inherently provides very high-sensitivity detection, potentially extendable to the quantum noise limit, therefore a signal-to-noise ratio larger than 35 dB and a relatively high dynamic range (≈45 dB) were achieved in the experiment.

### QCL fabrication procedure

A resonant phonon single longitudinal mode THz QCL^20^ emitting at the wavelength of 76.3 μm (3.93 THz) with a surface plasmon waveguide, was grown by molecular beam epitaxy employing a GaAs/Al_0.15_Ga_0.85_As heterostructure on a nominally undoped GaAs substrate. The active region was engineered with two upper laser levels closely separated by about 1 meV energy. A 500 nm thick layer heavily doped (3.0 × 10^18^ cm^−3^, Si) defines the lower contact of laser. The active region is repeated 120 times and the growth ends with a heavily doped (5.0 × 10^18^ cm^−3^, Si) GaAs (200 nm) contact layer. Lasing at 3.8 THz with a threshold current density of 82 A/cm^2^ at 5 K was demonstrated. The maximum output power is achieved near 400 A/cm^2^, still 2.5–3 times above threshold. Lasing is observed in pulsed mode up to 70 K with a peak power level in excess of 30 mW at 10 K.

### Numerical simulations

The transcendental equation Eq. (2) for the frequency 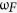 of the QCL in presence of optical feedback was numerically solved using a bisection method^31^ and the corresponding value of the carrier density Δ*N* was calculated using Eq. (1). Because of the existence of multiple solutions of Eq. (2) from moderate to strong feedback strength^18^, we choose to produce the SMI signal in Fig. 3c and Fig. 3d with the solution 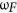 corresponding to the maximum gain mode, i.e. minimum Δ*N*.

The integrals in the expressions (6) and (7) for effective permittivities and associated complex reflectivities, were numerically evaluated using adaptive Gauss-Kronrod quadrature method^31^.

## Additional Information

**How to cite this article**: Mezzapesa, F. P. *et al.* Photo-generated metamaterials induce modulation of CW terahertz quantum cascade lasers. *Sci. Rep.* **5**, 16207; doi: 10.1038/srep16207 (2015).

## Supplementary Material

Supplementary Information
