# Corrigendum: Photo-generated metamaterials induce modulation of CW terahertz quantum cascade lasers

**DOI:** 10.1038/srep18547

**Published:** 2016-02-09

**Authors:** Francesco P. Mezzapesa, Lorenzo L. Columbo, Carlo Rizza, Massimo Brambilla, Alessandro Ciattoni, Maurizio Dabbicco, Miriam S. Vitiello, Gaetano Scamarcio

Scientific Reports 5: Article number: 1620710.1038/srep16207; published online 11092015; updated: 02092016

The original version of this Article contained a typographical error in the spelling of the author Alessandro Ciattoni which was incorrectly given as Alessardro Ciattoni. This has now been corrected in the PDF and HTML versions of the Article.

