# Supplementary material for: Photo-generated metamaterials induce modulation of CW terahertz quantum cascade lasers
Source: Sci Rep. 2015 Nov 9;5:16207. doi: 10.1038/srep16207 (PMC4637892; doi:10.1038/srep16207)
Supplement: Supplementary Information [file srep16207-s1.pdf]

Photo-generated metamaterials induce modulation of CW terahertz quantum cascade lasers

Francesco P. Mezzapesa[1,2], Lorenzo L. Columbo[1,2], Carlo Rizza[3,4], Massimo Brambilla[1,2], Alessardro Ciattoni[4], Maurizio Dabbicco[1,2], Miriam S. Vitiello[5], and Gaetano Scamarcio[1,2]

[1]*Dipartimento Interateneo di Fisica, Università degli Studi e Politecnico di Bari,via Amendola 173, I-70126 Bari (Italy)*

[2]*CNR-IFN UOS Bari, via Amendola 173, I-70126 Bari (Italy)*

[3]*Dipartimento di Scienza e Alta Tecnologia, Università dell'Insubria, via Valleggio 11, I-22100 Como (Italy)*

[4]*CNR-SPIN, via Vetoio 10, I-67100 L'Aquila (Italy)*

[5]*NEST, CNR - Istituto Nanoscienze and Scuola Normale Superiore, piazza San Silvestro12, I-56127 Pisa (Italy)*

Supplementary Information

Supplementary Discussion 1: Detailed discussion of homogenization theory for periodic photo-designed THz metamaterials.

In the context of metamaterials, the homogenization theory is a fundamental tool for giving an effective description of the electromagnetic behavior of complex heterogeneous subwavelength structures through the introduction of effective electromagnetic parameters (e.g. effective permittivity, effective permeability). Homogenization techniques have a long-standing tradition and many authors have developed different approaches in the past for predicting the electromagnetic effective response [1]. Recently, A. Ciattoni and C. Rizza have proposed a first-principles homogenization theory based on a multiscale technique and they have shown that the effective medium permittivity tensor and the first- and second-order tensors describing spatial dispersion can be evaluated by averaging suitable spatially rapidly varying fields, each satisfying electrostatic-like equations within the metamaterial unit cell [2]. Generally, the standard metamaterials are composed by subwalength material inclusions and they are characterized by the fact that the "microscopic" relative dielectric permittivity depends on a subwavelength scale only.

Here, our theoretical effort is to describe photo-designed metamaterials where the "microscopic" relative dielectric permittivity exhibits a continuous spatial variation and, in addition, it is characterized both by a fast (subwavelength) and a slow spatial modulation. In order to achieve this result, we have considered the theory reported in Ref.[2] (suitable for describing subwalength structures with a continuous spatial variation of the dielectric permittivity) in the very long-wavelength limit and in the situation where the "microscopic" dielectric relative permittivity depends on fast (subwavelength) and slow scales. We consider the Maxwell's equations $\nabla\times\mathbf{E}=i\omega_{THz}\mu_0\mathbf{H},\nabla\times\mathbf{H}=-i\omega_{THz}\varepsilon_0\varepsilon_{sc}\mathbf{E},$ where the time dependence is $e^{-i\omega_{THz}t}$. Following the multiscale approach of Ref.[2], we define the small dimensionless parameter $\eta=\Lambda/\lambda_{THz}\ll 1$, namely the ratio between the structure period Λ and the terahertz radiation wavelength λ_{THz} and, in addition, we suppose that the "microscopic" dielectric relative permittivity ε_{sc} is a 2D periodic function in the x-y plane and we set $\varepsilon(z,\mathbf{R}_\perp)=\varepsilon_{sc}(z,\mathbf{r}_\perp)$, where z is the standard spatial coordinate along the axis perpendicular to the air-semiconductor interface and $\mathbf{R}_\perp=\mathbf{r}_\perp/\eta$ ($\mathbf{r}_\perp=x\hat{\mathbf{e}}_x+y\hat{\mathbf{e}}_y,\mathbf{R}_\perp=X\hat{\mathbf{e}}_x+Y\hat{\mathbf{e}}_y$). Using a two-scale expansion for electromagnetic field amplitudes $\mathbf{A}=\mathbf{E},\mathbf{H}$, we set

$$\mathbf{A}=\bar{\mathbf{A}}_0(\mathbf{r})+\tilde{\mathbf{A}}_0(\mathbf{r},\mathbf{R}_\perp)+\eta\left[\bar{\mathbf{A}}_1(\mathbf{r})+\tilde{\mathbf{A}}_1(\mathbf{r},\mathbf{R}_\perp)\right], \quad (1)$$

namely the field amplitude are expanded up to the first-order in η where the overline and the tilde denote the spatial average over the unit cell and the rapidly varying zero mean residual, respectively. Noting that $\nabla_\perp\to\nabla_\perp+\eta^{-1}\nabla_{\mathbf{R}_\perp}$, where $\nabla_\perp=\hat{\mathbf{e}}_x\partial_x+\hat{\mathbf{e}}_y\partial_y$ and $\nabla_{\mathbf{R}_\perp}=\hat{\mathbf{e}}_x\partial_X+\hat{\mathbf{e}}_y\partial_Y$, substituting Eq.(1) into Maxwell's equations, separating each order in η, we obtain equations for both the average and zero mean residual contributions. The average equations for the zero-th order are given by

$$\nabla\times\bar{\mathbf{E}}_0=i\omega_{THz}\mu_0\bar{\mathbf{H}}_0,\quad \nabla\times\bar{\mathbf{H}}_0=-i\omega_{THz}\varepsilon_0\left(\bar{\varepsilon}\bar{\mathbf{E}}_0+\overline{\tilde{\varepsilon}\tilde{\mathbf{E}}_0}\right), \quad (2)$$

where we have used $\varepsilon(z,\mathbf{R}_\perp)=\bar{\varepsilon}(z)+\tilde{\varepsilon}(z,\mathbf{R}_\perp)$ ($\bar{\varepsilon}$ and $\tilde{\varepsilon}$ are the average over the unit cell and the zero mean residual contribution, respectively) whereas, the equations for zero mean residual fields are

$$
\begin{aligned}
&\nabla_{\mathbf{R}_\perp} \times \tilde{\mathbf{E}}_0 = 0, \quad \nabla_{\mathbf{R}_\perp} \times \tilde{\mathbf{H}}_0 = 0, \\
&\nabla \times \tilde{\mathbf{E}}_0 + \nabla_{\mathbf{R}_\perp} \times \tilde{\mathbf{E}}_1 = i\omega_{THz}\mu_0 \tilde{\mathbf{H}}_0, \\
&\nabla \times \tilde{\mathbf{H}}_0 + \nabla_{\mathbf{R}_\perp} \times \tilde{\mathbf{H}}_1 = -i\omega_{THz}\varepsilon_0 \left(\varepsilon \tilde{\mathbf{E}}_0 + \tilde{\varepsilon} \bar{\mathbf{E}}_0 - \overline{\tilde{\varepsilon} \tilde{\mathbf{E}}_0} \right),
\end{aligned} \qquad (3)
$$

Applying the operator $\nabla_{\mathbf{R}_\perp} \cdot$ to both the third and the fourth of Eqs.(3), using the identity $\nabla_{\mathbf{R}_\perp} \cdot (\nabla \times \mathbf{A}) = -\nabla \cdot \left(\nabla_{\mathbf{R}_\perp} \times \mathbf{A} \right)$ and exploiting the first and second of Eqs.(3), these two equations become

$$
\begin{aligned}
&\nabla_{\mathbf{R}_\perp} \cdot \tilde{\mathbf{H}}_0 = 0, \\
&\nabla_{\mathbf{R}_\perp} \cdot \left(\varepsilon \tilde{\mathbf{E}}_0 + \tilde{\varepsilon} \bar{\mathbf{E}}_0 \right) = 0,
\end{aligned} \qquad (4)
$$

Considering the first and the second of Eqs.(3) and the first of Eqs.(4), and using the fact that $\tilde{\mathbf{H}}_0$ and $\tilde{\mathbf{E}}_0$ have vanishing mean value, we get

$$
\begin{aligned}
&\tilde{\mathbf{H}}_0 = 0, \ \tilde{E}_{0z} = 0, \\
&\tilde{E}_{0x} = \partial_X \mathbf{f} \cdot \bar{\mathbf{E}}_0, \quad \tilde{E}_{0y} = \partial_Y \mathbf{f} \cdot \bar{\mathbf{E}}_0,
\end{aligned} \qquad (5)
$$

where the components of the vector $\mathbf{f} = f_X \hat{\mathbf{e}}_x + f_Y \hat{\mathbf{e}}_y$ satisfy the equations

$$
\nabla_{\mathbf{R}_\perp} \cdot \left(\varepsilon \nabla_{\mathbf{R}_\perp} f_X \right) = -\partial_X \varepsilon, \quad \nabla_{\mathbf{R}_\perp} \cdot \left(\varepsilon \nabla_{\mathbf{R}_\perp} f_Y \right) = -\partial_Y \varepsilon. \qquad (6)
$$

Inserting the electric field components of Eqs.(5) into Eqs.(2), the average equations become

$$
\nabla \times \bar{\mathbf{E}}_0 = i\omega_{THz}\mu_0 \bar{\mathbf{H}}_0, \quad \nabla \times \bar{\mathbf{H}}_0 = -i\omega_{THz}\varepsilon_0 \varepsilon^{(eff)} \bar{\mathbf{E}}_0, \qquad (7)
$$

where

$$
\varepsilon^{(eff)} = \begin{pmatrix} \bar{\varepsilon} + \overline{\varepsilon \partial_X f_X} & \overline{\varepsilon \partial_X f_Y} & 0 \\ \overline{\varepsilon \partial_Y f_X} & \bar{\varepsilon} + \overline{\varepsilon \partial_Y f_Y} & 0 \\ 0 & 0 & \bar{\varepsilon} \end{pmatrix} \qquad (8)
$$

Next, we consider the one-dimensional case where the photo-induced profile is $\varepsilon(z, X)$. The dielectric profile and its inverse are represented by Fourier series

$$
\varepsilon(z, X) = \sum_{n=-\infty}^{+\infty} a_n(z) e^{ink_0 X}, \quad \varepsilon^{-1}(z, X) = \sum_{n=-\infty}^{+\infty} b_n(z) e^{ink_0 X} \qquad (9)
$$

where $k_0 = 2\pi / \lambda_{THz}$. By using Fourier representations of Eqs.(9), we solve Eqs.(6) and we get

$$f_X(z,X) = -\frac{i}{k_0 b_0}\sum_{n=-\infty}^{+\infty}\frac{b_n(z)}{n}e^{ink_0X}, \quad f_Z(z,X)=0, \qquad (10)$$

so that, substituting Eqs.(10) in the expression for the effective dielectric permittivity tensor of Eq.(8), noting that $a_0 = \overline{\varepsilon}, \quad b_0 = \overline{\varepsilon^{-1}}$, we finally get,

$$\varepsilon_{11}^{(eff)} = \left(\overline{\varepsilon^{-1}}\right)^{-1}, \quad \varepsilon_{22}^{(eff)} = \varepsilon_{33}^{(eff)} = \overline{\varepsilon}, \qquad (11)$$

which reproduce the result of the standard effective medium theory [1] even with the substantial difference that these quantity parametrically depend on z. As a consequence, in the considered geometry depicted in Fig.1, we obtain

$$\begin{aligned}\varepsilon_{\parallel}(z) &= \varepsilon_{22}^{(eff)}(z) = \varepsilon_{33}^{(eff)}(z) = \frac{1}{\Lambda}\int_0^{\Lambda} dx\varepsilon_{sc}(x,z),\\ \varepsilon_{\perp}(z) &= \varepsilon_{11}^{(eff)}(z) = \frac{1}{\Lambda}\int_0^{\Lambda} dx\varepsilon_{sc}^{-1}(x,z),\end{aligned} \qquad (12)$$

coinciding with Eqs.(6) in the manuscript.

Supplementary Discussion 2: Complex reflection coefficients for photo-designed THz metamaterials

In order to obtain the analytical expression of Eq.(7) of the main text, containing the Fresnel reflection coefficients of a photo-designed THz metamaterial, we consider the situation where the THz plane wave normally impinge on the air-semiconductor interface. Inside the semiconductor slab, the electric field is given by $\overline{\mathbf{E}}_0 = \mathbf{U}_+ e^{ik_z z} + \mathbf{U}_- e^{-ik_z z}$ $[\mathrm{k_z} = (\omega_{\mathrm{THz}}/\mathrm{c})\sqrt{\mathrm{Re}(\varepsilon_\infty)}]$ ($\mathbf{U}_\pm = U_{\pm x}\hat{\mathbf{e}}_x + U_{\pm y}\hat{\mathbf{e}}_y$ are the (+) forward and (-) backward electric field amplitudes) and exploiting the standard slowly varying approximation, we get

$$\begin{aligned}\pm 2\mathrm{i}\mathrm{k_z}\partial_z \mathrm{U}_{\pm x} &= \left[\mathrm{k_z^2} - \mathrm{k_0^2}\varepsilon_{\parallel}(\mathrm{z})\right]\mathrm{U}_{\pm x},\\ \pm 2\mathrm{i}\mathrm{k_z}\partial_z \mathrm{U}_{\pm y} &= \left[\mathrm{k_z^2} - \mathrm{k_0^2}\varepsilon_{\perp}(\mathrm{z})\right]\mathrm{U}_{\pm y}.\end{aligned} \qquad (13)$$

Equations (12) yield

$$\mathrm{U}_{\pm x}(\mathrm{z}) = \mathrm{U}_{\pm x}(0)\,\mathrm{e}^{\mp\frac{\mathrm{i}}{2\mathrm{k_z}}\int_0^{\mathrm{z}}\mathrm{dz'}\left[\mathrm{k_z^2}-\mathrm{k_0^2}\varepsilon_{\parallel}(\mathrm{z})\right]}, \quad \mathrm{U}_{\pm y}(\mathrm{z}) = \mathrm{U}_{\pm y}(0)\,\mathrm{e}^{\mp\frac{\mathrm{i}}{2\mathrm{k_z}}\int_0^{\mathrm{z}}\mathrm{dz'}\left[\mathrm{k_z^2}-\mathrm{k_0^2}\varepsilon_{\perp}(\mathrm{z'})\right]} \qquad (14)$$

By using Eqs.(14) and imposing the continuity of the tangent components of the electric and magnetic fields at the air- semiconductor interfaces, we obtain the Fresnel reflection coefficients reported in Eqs.(7) in the manuscript.

Supplementary References

1. Cai, W. , Shalaev, V. Optical Metamaterials: Fundamentals and Applications (Springer, 2010).
2. Ciattoni, A., Rizza, C. Nonlocal homogenization theory in metamaterials: Effective electromagnetic spatial dispersion and artificial chirality, *Phys. Rev. B* **91**, 184207 (2015).
